# Cargo exchange between human and bacterial extracellular vesicles in gestational tissues: a new paradigm in communication and immune development

**DOI:** 10.20517/evcna.2024.21

**Published:** 2024-06-18

**Authors:** Emmanuel Amabebe, Awanit Kumar, Madhuri Tatiparthy, Ananth Kumar Kammala, Brandie D. Taylor, Ramkumar Menon

**Affiliations:** Department of Obstetrics and Gynecology, The University of Texas Medical Branch at Galveston, Galveston, TX 77555, USA.

**Keywords:** Extracellular vesicles, placenta, fetal membranes, feto-maternal interface, immune priming, outer membrane vesicles

## Abstract

Host-bacteria and bacteria-bacteria interactions can be facilitated by extracellular vesicles (EVs) secreted by both human and bacterial cells. Human and bacterial EVs (BEVs) propagate and transfer immunogenic cargos that may elicit immune responses in nearby or distant recipient cells/tissues. Hence, direct colonization of tissues by bacterial cells is not required for immunogenic stimulation. This phenomenon is important in the feto-maternal interface, where optimum tolerance between the mother and fetus is required for a successful pregnancy. Though the intrauterine cavity is widely considered sterile, BEVs from diverse sources have been identified in the placenta and amniotic cavity. These BEVs can be internalized by human cells, which may help them evade host immune surveillance. Though it appears logical, whether bacterial cells internalize human EVs or human EV cargo is yet to be determined. However, the presence of BEVs in placental tissues or amniotic cavity is believed to trigger a low-grade immune response that primes the fetal immune system for ex-utero survival, but is insufficient to disrupt the progression of pregnancy or cause immune intolerance required for adverse pregnancy events. Nevertheless, the exchange of bioactive cargos between human and BEVs, and the mechanical underpinnings and health implications of such interactions, especially during pregnancy, are still understudied. Therefore, while focusing on the feto-maternal interface, we discussed how human cells take up BEVs and whether bacterial cells take up human EVs or their cargo, the exchange of cargos between human and BEVs, host cell (feto-maternal) inflammatory responses to BEV immunogenic stimulation, and associations of these interactions with fetal immune priming and adverse reproductive outcomes such as preeclampsia and preterm birth.

## INTRODUCTION

Extracellular vesicles (EVs) are lipid bilayer, nano-sized, non-replicating (non-nucleated) subcellular particles released by all cells to facilitate cell-free intercellular communication and transport of molecules^[1-3]^. EVs promote cell-to-cell interaction by transferring diverse bioactive molecules and genetic information^[4]^. EVs represent the real-time snapshot of the parent cells’ physiologic or pathophysiologic state, and they protect their cargo (DNA, RNA, proteins, lipids, and metabolites) from degradation^[5-10]^. Hence, EVs serve as a valuable source of circulating indicators of the physiologic and pathologic state of a tissue that can be used as biomarkers or to develop targeted therapies^[4-10]^. EVs, as paracrine signalers, can deliver their cargo and elicit a response. Often, EVs interact with receptors on their target cells and trigger intracellular signaling via surface ligands^[11,12]^. For example, receptors expressed on EVs can engage in a ligand-receptor interaction with a cell expressing appropriate receptors, where cell signaling can be triggered. EVs can also signal through intracellular target molecules^[13,14]^.

Like humans, prokaryotic organisms like bacteria also produce vesicles referred to as outer membrane vesicles (OMVs), primarily from Gram-negative bacteria or, in general, bacterial extracellular vesicles (BEVs)^[15]^. Both human and bacterial-derived EVs are widely studied, although often exclusively. Human body sites (mouth, gut, skin, respiratory, and genital tracts), where host cells co-exist with bacterial species (microbiota) in a dynamic mutualistic relationship (microbiome)^[2,16-24]^, are sources of EVs from both humans and microbes. The host-bacteria and bacteria-bacteria interactions at such niches can be mediated by the exchange of biomolecules between both cell types, some of which are packaged and transported by EVs in a paracrine fashion^[2,16,25,26]^. Some researchers believe that EVs are the main communication pathway between human cells and the microbiota^[27]^ because EVs guarantee host-microbiome interaction without direct contact and in a bidirectional fashion^[24,28-30]^. Importantly, this EV-mediated communication channel is utilized or exploited by both commensal and infectious species^[31]^.

Accordingly, our group recently reported the presence of BEVs in placental tissues and postulated that the human placenta harbors BEVs secreted by endogenous commensal microbes or microbes from the individual’s environment (air, water, or food)^[26]^. However, the exchange of biomolecular cargos between human and bacterial EVs, and the mechanical underpinnings and health implications of such interactions, especially in pregnancy, are still understudied. Therefore, this review highlights the putative mechanisms and potential immunologic, physiologic and pathologic, biomarker, and therapeutic implications of cargo exchange between human and bacterial EVs. With a focus on the feto-maternal interface, we discussed how human cells take up BEVs and whether bacterial cells take up human EVs or their cargo, the exchange of cargos between human and BEVs, host cell (feto-maternal) inflammatory responses to BEV immunogenic stimulation, and associations of these interactions with fetal immune priming and adverse reproductive outcomes such as preeclampsia and preterm birth.

### Physiological role of human EVs

Besides direct cellular contacts, multicellular organisms, including humans, employ secreted molecules packaged in EVs to mediate physiological and pathological activities^[11,32,33]^. Depending on their origin, size, biosynthesis, cargo contents, tissue tropism and function, human EVs are classified into exosomes (30-160 nm), microvesicles (MVs, 100-1,000 nm), and apoptotic bodies (~50-5,000 nm)^[3,6,34-39]^. Exosomes are formed from the classic endocytic pathway in multivesicular bodies (MVBs). In contrast, MVs and apoptotic bodies are secreted into the extracellular space by direct budding of the plasma membrane of live and dying (apoptotic) cells, respectively^[37,38]^. Other human EVs include microparticles, oncosomes, prostasomes, and migrasomes^[3,6,11,39-44]^.

The secretion of EVs is mediated by endosomal sorting complexes required for transport (ESCRT) pathway proteins and their homologs, and ESCRT-independent pathways^[42-44]^. Furthermore, EVs express specific *transmembrane or lipid-bound extracellular* and *cytosolic* protein markers [Table 1]^[3,34,40,43,45-59]^. Generally, exosomes are enriched in TSG101, ALIX, or HSP70 when the intraluminal vesicles (ILV) are formed through the ESCRT-dependent pathway. However, the tetraspanins (CD9, CD63, and CD81) are enriched when the vesicles are formed via the ESCRT-independent pathway^[60-65]^. Documentation of these protein markers [Table 1] is part of the minimum criteria for reporting studies involving EV isolation and characterization^[3,40]^.

**Table 1:** Typical human extracellular vesicle protein markers

**Transmembrane or lipid-bound extracellular**	**Cytosolic**
Tetraspanins: CD9, CD63, CD81, CD82	Tumor susceptibility gene 101 (TSG101)
Major histocompatibility complex (MHC) class I (HLA-A/B/C)	Flotillin-1 and 2 (*FLOT1/2*)
Epithelial cell adhesion molecule (EpCAM)	Programmed cell death 6 interacting protein (ALIX)
Platelet endothelial cell adhesion molecule-1 (PECAM-1)	Vacuolar protein sorting-associated protein 4 (VPS4A/B)
Intercellular adhesion molecule-1 (ICAM1)	Arrestin domain-containing protein 1 (ARRDC1)
Heparan sulfate proteoglycans, including syndecans	Annexins (*ANXA*)
Integrins (ITGA/ITGB)	Caveolins
	RHOA
	ADP-ribosylation factor 6 (ARF6)
	Syntenin
	Heat shock proteins: HSC70 (*HSPA8*), HSP70 *(HSPA1A)*, and HSP84 (*HSP90AB1*)
	Rabs (Ras-related protein GTPases)
	Tau [microtubule-associated proteins (MAPT), neurons]

Humans employ EVs, especially exosomes, in several physiological processes, including cell growth, proliferation and differentiation, angiogenesis, immune responses, cellular signaling, migration, and metabolism. In pregnancy, an area where the authors of this review are primarily focused, EVs are involved in implantation^[66,67]^, feto-placental growth^[68-70]^, feto-maternal communication^[29,30]^, pregnancy immune homeostasis^[71-73]^, and most importantly, function as signalers of parturition between the fetus and the mother^[35,74-77]^. They are also indicated in pathological conditions, including autoimmune and neurodegenerative diseases, cancer progression, cardiovascular, respiratory, metabolic, and neurological disorders, infectious diseases, and host-bacteria interactions^[13,26,36,39,42,78-82]^.

#### Cargo packaging in human (eukaryotic) EVs

EVs package their cargos through ESCRT-dependent or -independent pathways^[37,38,80]^. Both pathways are believed to work in synergy, which may explain how exosomes can carry unique contents different from the parental cell^[38,80]^.

*Proteins*: The sorting of proteins into EVs follows specific ESCRT, tetraspanins, and lipid-dependent mechanisms^[37,38,80]^. The packaging of ESCRT proteins such as ALIX and TSG101, along with tetraspanins such as CD63, in EVs indicates the biogenesis as well as the selective protein sorting machinery present in EVs^[37,38,44,80]^.

The ESCRT is composed of four multi-protein complexes (ESCRT-0, -I, -II, and –III) and their accessory proteins, particularly the AAA ATPase VPS4, VTA1, and ALIX, which successively recognize and load protein cargos into ILVs^[80,83]^. ESCRT facilitates the loading of ubiquitinated proteins into ILVs by recognizing the ubiquitin tags in the proteins’ lysine residue(s)^[37,38,44,60,80]^. For example, soluble *Mycobacterium tuberculosis* proteins are transported into exosomes after ubiquitination^[84]^. The early-acting complexes (ESCRT-0, -I, -II) contain ubiquitin-binding domains and bind to and load ubiquitinated proteins into EVs. At the same time, the late-acting ESCRT-III and VPS4 terminate EV formation and budding^[44,85]^. ALIX can also load proteins, for example, protease activator receptor 1 and purinergic receptor P2Y1, into developing EVs in a ubiquitin-independent manner^[86,87]^.

In addition to *ubiquitination,* selective protein cargo sorting in EVs is also mediated by other post-translational modifications (PTMs), including the addition of proteins such as small ubiquitin-like modifiers (SUMO, *SUMOylation*), NEDD8 (*NEDDylation*), ISG15 (*ISGylation*), FAT10 (FAT10ylation)^[37,38,88-90]^, and removal of ubiquitin (*Deubiquitination*)^[80]^. Other PTMs that can sort proteins into EVs include phosphorylation, acetylation, myristoylation, glycosylation, citrullination, oxidation, prenylation, palmitoylation, amidation, biotinylation, deamination, formylation, glycation, hydroxylation, methylation, farnesylation, glutathionylation, geranylgeranylation, mono-ADP-ribosylation, GPI-anchor, WW domain and coiled-coil domain^[37,38,80,90]^.

These PTMs regulate the structure, subcellular localization (e.g., sorting into EVs), and function of the protein in a context-specific manner^[91,92]^. However, they may not be often observed in all molecules or cell types^[37]^. For example, besides ubiquitination, HSC71, HSP90, 14-3-3ε, C20, and pyruvate kinase type M2 (PKM2) may drive the sorting of MHC II cargo in dendritic cell (DC)-derived exosomes^[93]^. This shows that the sorting of MHC II into exosomes of DCs is not dependent on MHC II ubiquitination in contrast to the sorting of MHC II at MVBs destined for degradation by lysosomal hydrolases^[60,94,95]^. Therefore, the content of EVs does not always mirror the protein composition of the cells of origin, and this does not occur randomly^[37]^. However, the EV cargo changes reflect the cell's physiological or pathological state^[96]^.

*RNA:* Different types of RNA species -small and long non-coding RNAs, miRNAs, mRNAs, siRNA, and structural RNAs are actively and passively packaged into EV compartments^[97-99]^ via six different pathways: (1) RNA-binding proteins (RBP) (e.g., heterogeneous nuclear ribonucleoprotein (hnRNP) and SYNCRIP) and miRNA motifs (EXO-motifs); (2) 3’ miRNA sequence-dependent pathway; (3) miRNA-induced silencing complex (miRISC)-related pathway; (4) other RBPs-related pathways (e.g., YBX-1, Ago2, MVP, MEX3C, and La protein); (5) membrane proteins-dependent pathway (e.g., Caveolin-1, VPS4A, and nSMase2); and (6) lipid raft-related pathway (based on the mechanism of EV biogenesis)^[37,38,80,100-102]^. The binding and sorting of miRNAs into EVs by various RBPs and membranous proteins are summarized in the review by Groot and Lee^[100]^.

*DNA*: The majority of EV-associated DNA is located on the surface of the vesicle, while the gDNA and mtDNA exist inside the vesicle, protected from digestion by DNase^[103]^. gDNA is believed to be packaged into EVs from micronuclei (MN), which are cytoplasmic structures derived from unstable nuclei and surrounded by nuclear membrane^[104,105]^. As shown in cancer cells, the unstable envelope of MN easily breaks down during cell division, releasing the gDNA and other nuclear contents. CD63 interacts with the disrupted MN and its contents to shuttle the DNA into exosomes referred to as nExo^[105-107]^. The ESCRT proteins can also facilitate the loading of DNA into small EVs, while ARF6 and RhoA mediate DNA loading into large EVs (MVs) from the cytosol^[105,108]^. Cell-free DNA (cfDNA) released by dead cells can also be packaged into EVs by sticking on the surface of released EVs^[106]^. However, packaging of mtDNA and other DNA types occurs through yet unknown mechanisms^[105,106,109]^, or through pathways similar to gDNA loading^[108]^.

*Lipids*: EVs are enriched in lipids, particularly the plasma membrane phosphatidylserine, sphingomyelin, and ceramide, and to a lesser extent, phosphatidylcholine, phosphatidylinositol, phosphatidylethanolamine, and phosphatidylglycerol^[110,111]^. Sorting of lipids and proteolipids in EVs is suggested to be associated with the yield and size of the EVs but independent of protein sorting^[111]^ and can be mediated by lipid raft domains^[38,112-114]^.

Cargo sorting in MVs depends on plasma membrane oligomerization^[37]^. Plasma membrane anchors target cytoplasmic proteins into MVs and to the budding site^[115]^. Like exosomes, MVs can also package proteins via the ESCRT-dependent pathway. For example, the binding of ARRDC1 and a component of ESCRT-I (TSG101) facilitates the loading of proteins into MVs and subsequent exocytosis via Gag-mediated budding^[37,54,116]^.

In summary, packaging of EV cargo (DNA, RNA, proteins, lipids) is a selective rather than random process mediated by selective ESCRT-dependent and -independent mechanisms. Understanding the mechanistic processes is important as the pathways of EV biogenesis, cargo sorting, and packaging considerably impact the functioning of an EV, namely, the determination of its targeted destination, cargo delivery, and recipient cell’s function^[37,38,80,100]^. For example, genetic deletion of CD81 hinders/reduces the incorporation/presence of the Rac gene (Rho family of GTPases) within EVs^[117]^. DCs isolated from CD9 knockout mice also exhibit decreased EV release, and CD9 influences the Wnt (Wingless-related integration site) signaling pathway by modulating the EV packaging of β-catenin^[118]^. This underscores a significant role for tetraspanins in EV biogenesis^[119]^. More information on the role of tetraspanins in packaging EVs with specific cargo has been published^[65,120,121]^.

### Physiologic role of bacterial EVs

**Figure 1:**
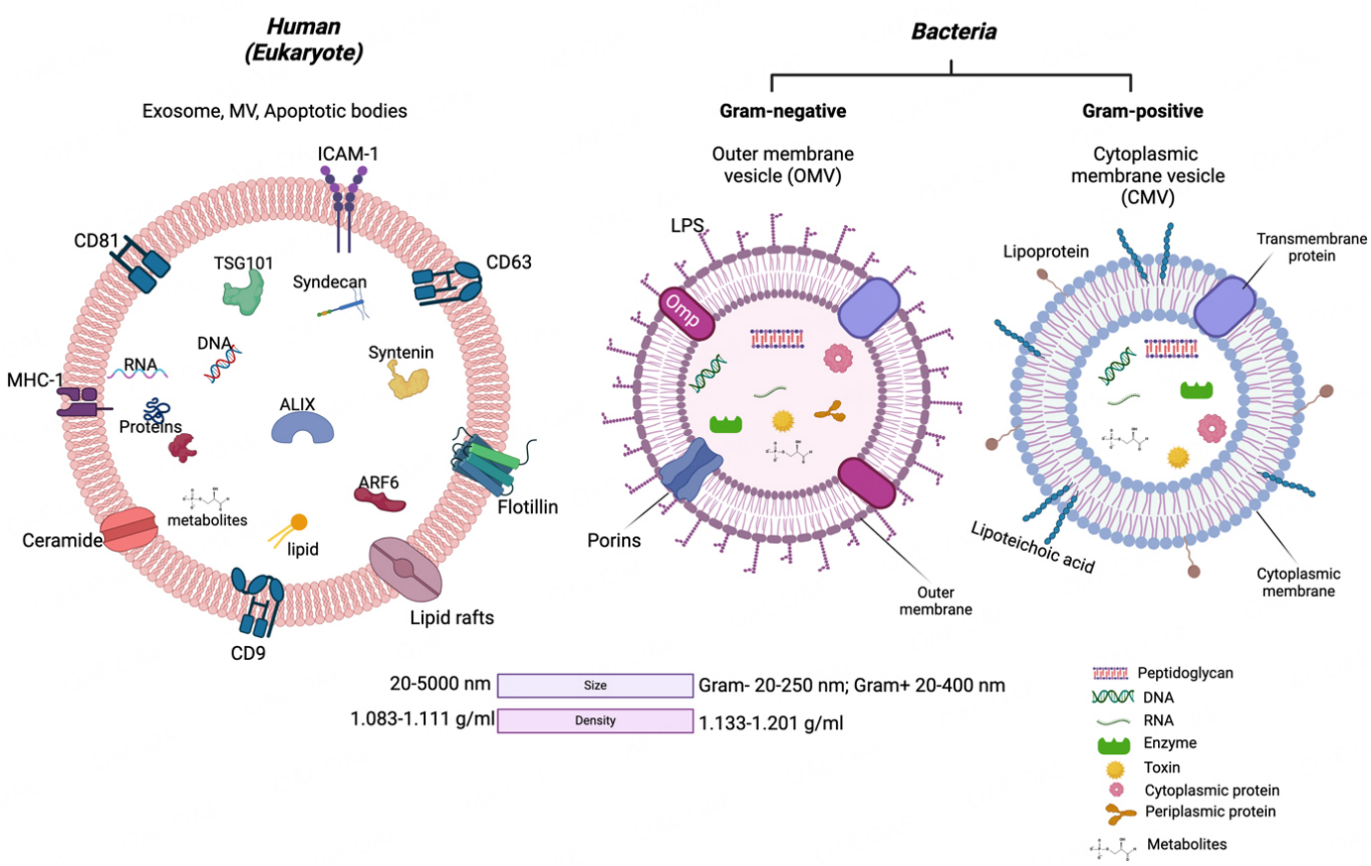
Comparison of human and bacterial-derived extracellular vesicles. ARF6: ADP ribosylation factor 6; ESCRT proteins (TSG101, ALIX); ICAM-1: intercellular adhesion molecule 1; LPS: lipopolysaccharide; Omp: outer membrane protein; MHC-1: major histocompatibility complex-1; Nuclei acids (DNA, RNA); Tetraspanins (CD9, CD63, CD81). Density data^[130]^. Created with BioRender.com.

In response to their environment, both pathogenic and commensal bacterial species secrete EVs that are similar in size to EVs derived from eukaryotic cells [Figure 1]^[2,4,23,122-130]^. Gram-positive and Gram-negative bacteria produce EVs that contain components of the parent cells^[2,4,131]^. An extensive comparison between Gram-negative and Gram-positive BEVs is published^[2,4,25]^.

BEVs from Gram-negative bacteria, as mentioned above, are called OMVs and are released by blebbing the bacterial outer membrane. OMVs (~20-250 nm) contain both periplasmic and cytoplasmic components [Figure 1]. Gram-negative bacteria also undergo explosive cell lysis to generate outer-inner membrane vesicles (O-IMV)^[25,132]^. Gram-positive bacteria form cytoplasmic membrane vesicles (CMVs, ~20-400 nm) by budding and shedding their cytoplasmic membrane, which eventually crosses the hydrolyzed peptidoglycan cell wall. CMVs carry cytosolic substances [Figure 1]^[25,132,133]^. Additionally, both Gram-negative and Gram-positive bacteria form tube-shaped membranous structures (50-70 nm) that connect the periplasms of cells within biofilms^[25,124,132,134]^. The differences between OMVs, CMVs, and eukaryotic (human) EVs are illustrated in Figure 1.

BEVs mediate bacteria-bacteria and bacteria-host interactions, promoting healthy and pathological conditions^[2,134]^. BEVs facilitate quorum sensing, promoting communication and coordinating group behavior among the species^[135,136]^. With the aid of diverse biomolecules transported as cargo, BEVs are also involved in bacterial competition and survival, adhesion, invasion of host tissue and infection, biofilm formation, resistance to antibiotics and other environmental stressors, host immune response and evasion, and determination of cell fate (autophagy)^[4,27,34,79,122,128,137-143]^.

Furthermore, BEVs promote the transfer of microbial genetic material (horizontal gene transfer (HGT) and the exchange of toxins and virulence factors^[4,122,144,145]^. BEVs contain a myriad of biomolecules, including microbial genetic components (luminal and surface-associated DNA, sRNA, mRNA, miRNA), metabolites, virulence factors (phospholipase C, alkaline phosphatase, esterase lipase, serine protease), and toxins (cholera, adenylate cyclase, cytolethal distending, VacA, PagJ, PagK1)^[2,122,123,144]^. They also contain proteins and glycoproteins that aid bacterial adhesion, invasion, survival, and immune evasion (OmpA, adhesin/invasin, plasma binding proteins, cytotoxic necrotizing factor 1, hemin-binding protein C), antibiotic resistance (β-lactamase, cephalosporinases, BlaZ, enzyme L5, transferring carbapenemase gene [OXA-24 gene], colistin, polymyxin B, ampicillin EV entrapment, penicillin-binding proteins, and multidrug efflux protein), and biofilm formation (alkaline protease, PrpL, CdrA)^[2,4,26,122,125,146,147]^.

Although with limitations that are being addressed^[148]^, native and bio-engineered EVs derived from pathogenic bacteria are also potential sources of vaccines ^[2,4,42]^. For instance, vaccination against tumors and intracellular viruses has been demonstrated using EVs derived from hypervesiculating *Salmonella typhimurium* that induce antigen-specific CD8+ T cell responses^[149]^. Similarly, EVs from *S. aureus* induce specific humoral and cellular immune responses. Mice vaccinated with *S. aureus*-derived EVs were protected from pneumonia and mortality induced by the administration of sublethal and lethal doses of *S. aureus,* respectively^[150]^. Subsequently, genetically engineered *S. aureus*-derived EVs that expressed detoxified cytolysins elicited cytolysin-neutralizing antibodies in mice that protected the animals in a lethal sepsis model^[133]^. Additionally, EVs from *S. aureus* coated with indocyanine green-labeled mesoporous silica nanoparticles were protective against drug-resistant *S. aureus* infection^[151]^. Because Gram-positive bacteria do not contain LPS, they could show better vaccine development prospects than Gram-negative bacteria^[152]^.

#### Cargo packaging in BEVs

This review will introduce some fundamental concepts of cargo loading in BEVs, and a detailed discussion on the mechanisms is not attempted here but can be seen in references^[153-161]^.

*Proteins*: The biological functions of EVs are determined by their protein cargo^[153]^. Studies have examined how BEV cargo is packaged using several strains of *S. aureus* to generate ideas for new interventions against pathogenic bacteria and develop drug delivery systems^[153]^. In *S. aureus,* it was shown that there is a selective cargo sorting process in BEVs^[153]^. The packaging of proteins in EVs was driven by abundance, charge, and subcellular localization^[153,162-164]^. Though more investigation is required, it is believed that EV cargo also contains components such as chaperones and protein secretion systems for the selection of proteins into EVs^[153]^.

This is reminiscent of a previous report that OMVs strictly sort their protein cargos by the special signal sequences (signal peptides) in the amino acid sequences^[23,154]^. The signal peptides guide proteins to their target vesicles which must possess the corresponding receptor on their membranes to recognize the signal peptides^[23,155,156,158]^. BEVs can also internalize foreign proteins via fusion to P49 protein expressed in *Shewanella vesiculosa* HM13 strain^[157]^.

Selective cargo sorting into BEVs also includes outer membrane lipid chaperones. Outer membrane lipid chaperones enriched with LPS carrying negatively charged O antigen chains (A-LPS) may play a critical role in selective cargo packaging due to their affinity for the overall charge^[159,165]^. This increased the selective packaging of virulence factors such as gingipains into *Porphyromonas gingivalis* OMVs^[159]^. Proteins associated with charged LPS are packaged in OMVs, while those associated with neutral LPS (O-LPS) are localized in the outer membrane^[158,159,161]^. Similarly, the lipid composition of *Streptococcus pyrogens* membrane was responsible for the selective enrichment of specific proteins and RNA species^[160]^.

*Nucleic acids*: Although the mechanism by which nucleic acids are packaged by OMVs remains unclear, DNA, mRNA, miRNA, and other non-coding RNAs may enter OMVs through a similar recognition of corresponding sequences^[166,167]^. EVs carry DNA both on the membrane surface and in the lumen, with most DNA located on the external surface of OMV^[145]^. Different forms of luminal DNA have been identified in OMVs secreted by *E. coli, Neisseria gonorrhoeae, Pseudomonas aeruginosa,* and *Haemophilus influenzae.* The luminal DNA retains its antigenicity even after treatment of the vesicle with DNase^[168]^.

*Lipids*: The lipid composition of Gram-negative and Gram-positive BEV are different. The most common lipids in Gram-negative BEV are phosphoglycerolipids. Other lipids found in Gram-negative BEV include lipoproteins, LPS, glycerolipids, and phospholipids. In Gram-positive bacteria such as *S. pyrogens*, the BEVs are abundant in phosphatidylglycerol and lack cardiolipin^[160]^.

### Cargo exchange between human and bacterial EVs

BEVs facilitate bacteria-host and bacteria-bacteria interactions with their cargos^[25,131]^ [Figure 1] that also deliver toxins and virulence factors into host cells^[131,169]^.

#### Uptake of BEVs by human (host) cells

**Figure 2:**
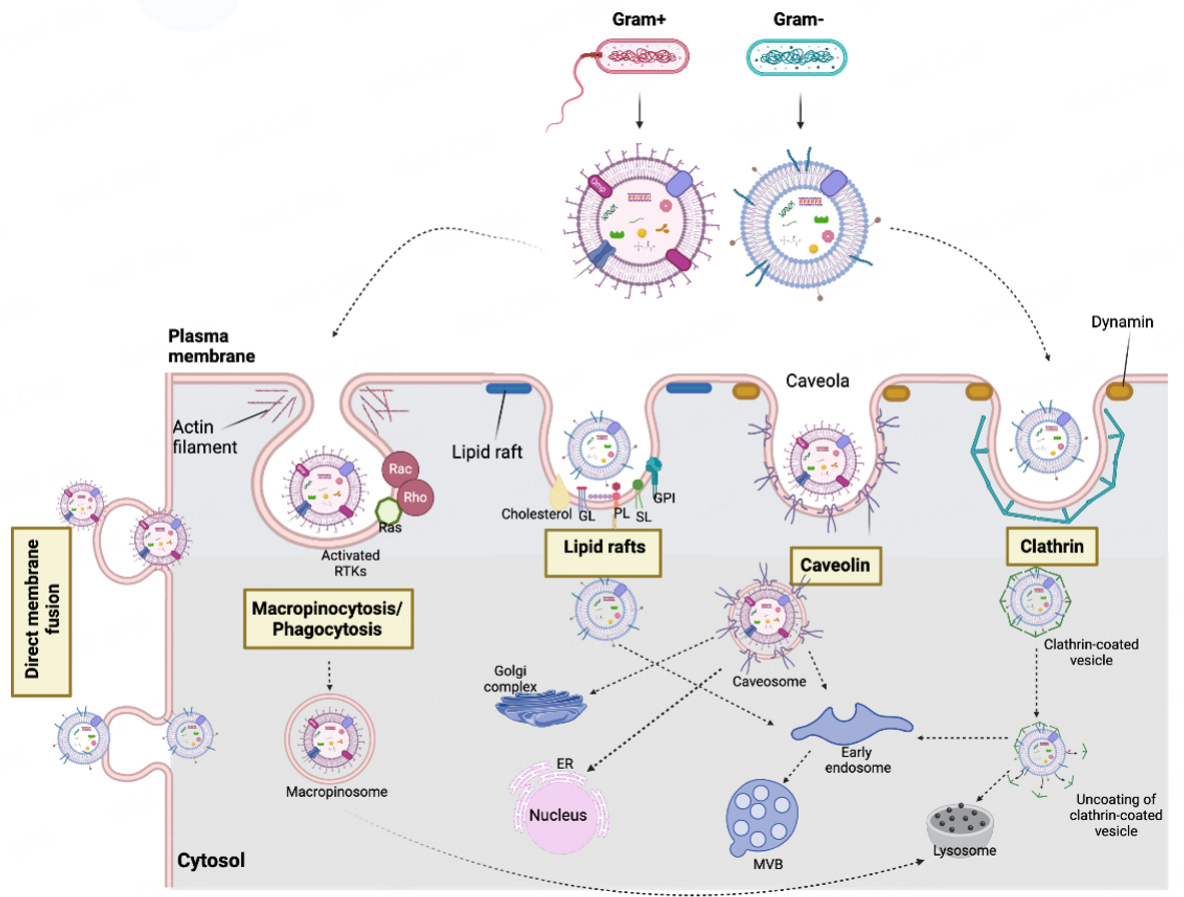
Mechanisms of uptake of bacterial extracellular vesicles by human (host) cells. BEVs enter human cells by macropinocytosis/phagocytosis, clathrin- and caveolin-mediated endocytosis, lipid rafts, and direct fusion with the plasma membrane. ER: endoplasmic reticulum; MVB: multivesicular bodies; GL: glycolipid; GPI: GPI-anchored proteins; PL: phospholipid; RTK: receptor tyrosine kinases; SL: sphingolipid. Created with BioRender.com.

After adhesion/binding of BEVs to host cells, non-phagocytic host cells internalize BEVs through the following mechanisms^[129,131,170]^: (1) macropinocytosis; (2) clathrin-mediated endocytosis; (3) caveolin-mediated endocytosis; (4) lipid raft-mediated endocytosis; and (5) direct membrane fusion^[25]^ [Figure 2 and Table 2].

**Table 2:** Mechanisms of BEV uptake by human (host) cells

**Mechanism/receptors**	**Cells**	**Bacterial species**
**Macropinocytosis/phagocytosis (actin-dependent)**		
	Macrophages	*P. aeruginosa* ^[171]^
N-WASP	Respiratory tract epithelial cells	*P. aeruginosa* ^[172]^
Rac-1 and Cdc42	Cervical and gingival epithelial cells	*P. gingivalis* ^[173]^
**Clathrin-mediated endocytosis**		
VacA	Gastric epithelial cells	*H. pylori* ^[174,175]^
Dynamin	Colorectal epithelial cells	EHEC^[176]^
Dynamin	Intestinal epithelial cells	*E. coli* ^[177]^
	Cervical epithelial cells and monocytes	*B. abortus* ^[178]^
**Caveolin-mediated endocytosis**		
Caveolin 1	Pharyngeal epithelial cells	*H. influenza* ^[179]^
CT-receptor	Intestinal epithelial cells	*V. cholerae* ^[180]^
Caveolin	Adrenal and intestinal epithelial cells	ETEC^[181]^
**Lipid raft-mediated endocytosis**		
PaAP	Lung epithelial cells	*P. aeruginosa* ^[182]^
	Intestinal epithelial cells	*C. jejuni* ^[183]^
Dynamin-independent	Intestinal epithelial cells	*V. cholerae* ^[184]^
Dynamin-dependent	Gastric epithelial cells	*H. pylori* ^[174,185]^
TLR2	Alveolar epithelial cells	*M. catarrhalis* ^[186]^
IgD BCR, TLR9 and TLR2	Tonsillar B cells	*M. catarrhalis* ^[187]^
	Cervical and gingival epithelial cells	*P. gingivalis* ^[173]^
		
	Cervical epithelial cells	*A. baumannii* ^[188]^
**Direct membrane fusion**		
	Respiratory tract epithelial cells	*P. aeruginosa* ^[172]^
	Cervical epithelial cells and gingival fibroblasts	*A. actinomycetemcomitans* ^[189,190]^
	Macrophages	*L. pneumophilia* ^[191]^

Cdc42: cell division control protein 42; CT-receptor: cholera toxin receptor; EHEC: enterohemorrhagic Escherichia coli; ETEC: enterotoxigenic Escherichia coli; IgD BCR: immunoglobulin D B cell receptor (BCR); N-WASP: Neuronal Wiskott-Aldrich Syndrome protein; PaAP: Pseudomonas aeruginosa aminopeptidase; Rac1: Ras-related C3 botulinum toxin substrate 1; VacA: vacuolating cytotoxin A.

*Actin-dependent macropinocytosis:* This is similar to phagocytosis but does not require direct contact with the internalized material^[192]^. It involves the rearrangement (polymerization) of actin filaments to form a ring under the cell membrane, which eventually envelopes a portion of the extracellular space by closing at the top^[131,193]^. Actin polymerization is driven by the receptor tyrosine kinases (RTKs) activation via Rac and Rho GTPases. The largest endocytic vesicles (> 1 μm) are produced by an actin-dependent pathway^[194]^, which could be N-WASP-mediated as in *Pseudomonas aeruginosa*^[172]^ or Rac1-regulated pinocytic pathway employed by *Porphyromonas gingivalis* OMVs that is independent of clathrin, dynamin, and caveolin^[173]^ [Figure 2 and Table 2].

*Clathrin-dependent endocytosis (CDE)*: This is the major vesicular trafficking pathway from the cell surface to the interior in mammalian cells^[195,196]^. Clathrin-coated pits that mature into clathrin-coated endocytic vesicles are assembled following the binding of a ligand (BEV) to a cell surface receptor^[195,197,198]^. CDE also requires adaptor protein complexes, a variety of endocytic accessory proteins, and phosphatidylinositol lipids^[195,196]^ and permits uptake of BEVs with a maximum size of 120 nm diameter. Using inhibitors of clathrin pit formation (Chlorpromazine) and dynamin (dynasore), this pathway was reported to be the preferred route of entry by OMVs from different strains of *E. coli*, *Helicobacter pylori*, and *Brucella abortus*, as well as free cytotoxic virulence factors including cholera and Shiga toxins and VacA of *H. pylori*^[131,175,177,178,199-201]^.

*Caveolin-mediated endocytosis*: Membrane invaginations (caveolae) are formed and internalized in a dynamin-dependent fashion^[192]^. The formation of caveolae is due to the enrichment of membrane lipid-raft domains with caveolin, cholesterol, and sphingolipids. Smaller OMVs (20-100 nm) from *E. coli*, *H. pylori*, *Moraxella catarrhalis*, *Vibro cholerae*, and *Haemophilus influenzae* are preferentially taken up by host cells via caveolin-mediated endocytosis^[179,180,186,202,203]^. The cholera toxin from *V. cholerae* and heat-labile enterotoxin LT1 contained in *E. coli*-derived vesicles bind to the glycosphingolipid GM1 that is found on membrane lipid rafts enriched with caveolin, facilitating their uptake via caveolin-enriched endocytic vesicles^[180,203]^. This mechanism is preferred by *E. coli*, *P. aeruginosa*, *Campylobacter jejuni*, *V. cholerae*, *S. typhimurium, H. influenzae* and some viruses^[204]^*,* as the pathogens avoid trafficking to lysosomes and subsequent degradation when they are internalized via caveolae compared to clathrin-coated membrane invaginations^[170]^. Although caveolin-mediated endocytosis is five times slower than the clathrin-mediated pathway, it facilitates the efficient delivery of cargo to the cytosol^[192,198,205]^.

*Lipid raft-mediated endocytosis*: Lipid rafts are highly organized and rigid cholesterol and sphingolipids-enriched plasma membrane domains that can internalize signaling molecules up to 90 nm^[154,173,179,184,188,192]^. OMVs derived from *P. aeruginosa*, *P. gingivalis*, *C. jejuni*, *A. baumannii*, *V. cholera*, and *Vibrio vulnificus* require lipid rafts for entry into host cells^[173,182-184,188,206]^.

*Direct membrane fusion*: As demonstrated for *P. aeruginosa*, *A. actinomycetemcomitans*, and *Legionella pneumophila*, OMVs also enter host cells by direct fusion with the host cell plasma membrane often at lipid raft domains^[172,189,191]^ [Figure 2 and Table 2].

The mechanism of uptake of BEVs by host cells is dependent on their size, content, biogenesis, bacterial growth rate, and the fate of vesicles after uptake, even when derived from the same bacterial species. Specific protein or lipid cargos of BEVs could guide them to a specific uptake route^[131,192]^. This advocates cell-specific EV uptake^[192]^. The route of uptake of BEVs, in turn, determines the delivery and fate of the vesicles and their cargo^[131]^.

#### Uptake of human EVs by bacterial cells

The mucosae of various human body sites harbor trillions of different bacterial strains that form respective microbiota, of which the oral, gut, and vaginal microbiotas are quintessential examples^[2,20-22,207,208]^. Bacteria present in these microbiotas produce BEVs involved in bacteria-bacteria and bacteria-host interactions^[2,134,207]^ that promote health or propagate infections^[209]^. For instance, HIV-1 bound to the OMVs of *P. gingivalis* (an oral bacteria) was able to enter nonpermissive human oral keratinocytes (HOK) cells via vesicle endocytosis [Figure 2] and cause infection^[210]^. On the other hand, BEVs from commensal intestinal bacteria - *Enterobacter cloacae* and *Bacteroides thetaiotaomicron*-reduced replication of norovirus during coinfection of RAW 264.7 macrophages by inducing the gene expression of IL-1β, IL-6, TNF-α, and IFN-γ^[211]^.

Although the mechanisms of uptake of BEVs by human host cells have been proposed as described above [Figure 2], whether the same mechanisms apply to the uptake of human EVs by bacterial cells remains unclear. Even BEV interactions with bacterial cells appear confined to their cargo, with vague data on the uptake of BEVs by bacterial cells. Meanwhile, we can speculate that bacterial cells may package human and BEVs and/or their cargo during host-bacteria and bacteria-bacteria interactions in various body microbiomes or sites distant from the microbiomes, including the feto-maternal interface. BEV-mediated bacteria-bacteria interaction is displayed succinctly in quorum sensing, antibiotic resistance, biofilm formation, and survival^[2]^. For example, BEV-mediated antibiotic resistance is exhibited by *Bacteroides* spp., a dominant genus in the gut and vaginal microbiota that is associated with bacterial vaginosis (BV) and premature birth^[20-22,212]^. *Bacteroides* BEVs contain cephalosporinases that hydrolyze the β-lactam rings in antibiotics, thereby protecting both pathogens and commensal bacteria from antibiotics of the β-lactam type^[213]^.

Recently, our group isolated and characterized BEVs from human placental tissues^[26]^. We posited that the BEVs probably reached the placenta through hematogenous spread from various maternal body sites harboring microbiomes. Furthermore, their presence may trigger a low-grade localized inflammation to prime an antigen response in the placenta. However, it is insufficient to cause an overt fetal inflammatory response that can lead to adverse pregnancy events^[26]^. Nevertheless, whether the BEVs contained human EV cargo and vice versa was not determined.

It is known that human EVs can take up free or EV-associated bacterial products, and BEVs may exchange their cargo with human cells or EVs. As the vagina is the most common route of bacterial colonization of the choriodecidual membranes and amniotic cavity^[214]^, it is pertinent for researchers to investigate whether part of the mechanisms of infectious and inflammatory adverse reproductive outcomes is mediated by human cells/EVs carrying bacterial products from the vagina or bacterial cells/EVs carrying human cargo that help them evade immune clearance.

### BEV-induced inflammatory responses

**Figure 3:**
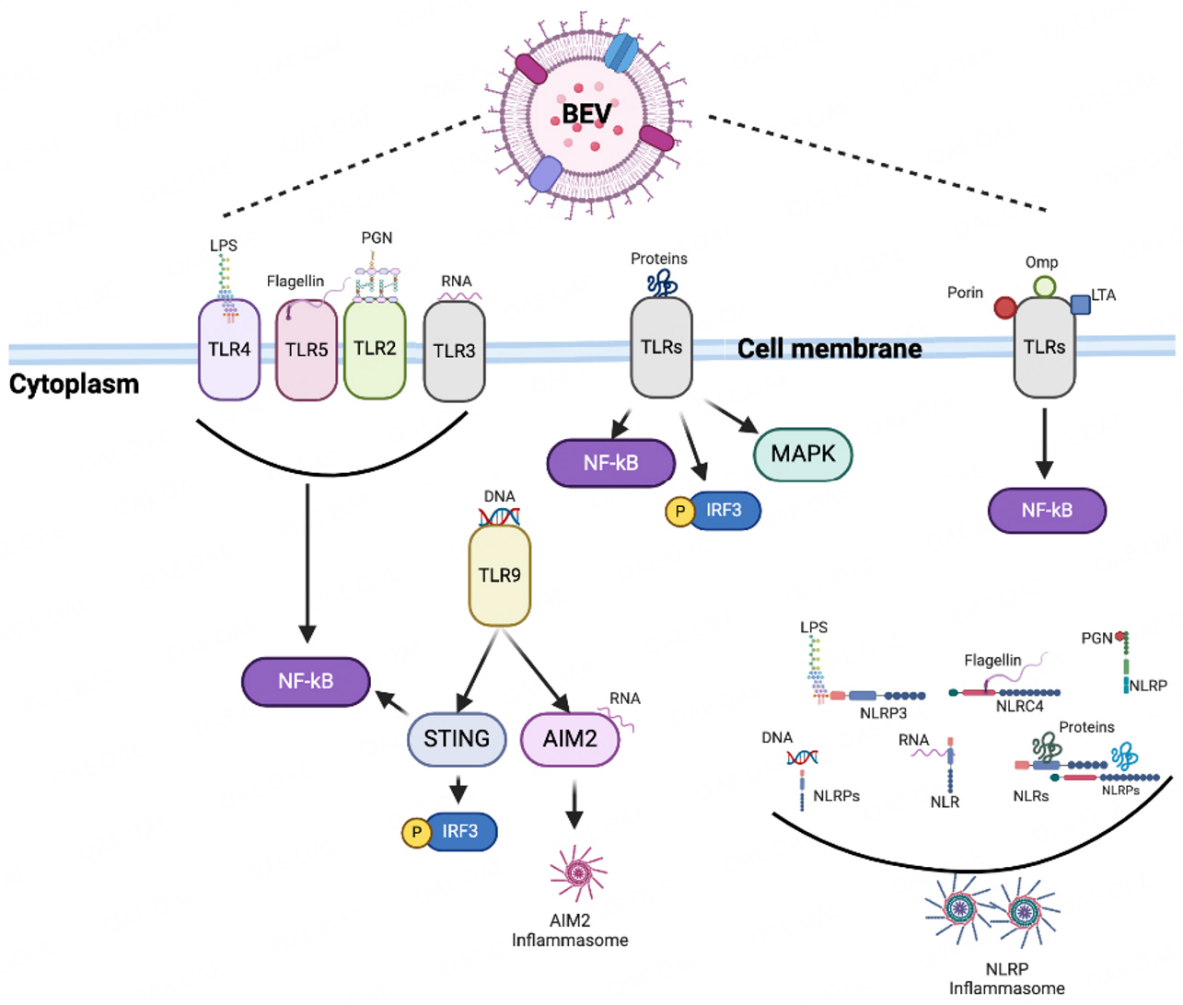
Bacterial extracellular vesicle (BEV) pathogen-associated molecular patterns (PAMPs) recognize surface membrane and cytoplasmic host cell pattern recognition receptors and activate downstream inflammatory signaling pathways. Omp, LTA, and porin only bind to TLRs on the cell membrane surface. At the same time, both surface membrane and cytoplasmic receptors recognize the other PAMPs. *AIM2*: absent in melanoma 2; *IRF*: interferon regulatory transcription factor; *LPS*: lipopolysaccharide; *LTA*: lipoteichoic acid; *MAPK*: mitogen-associated protein kinase; *NF-κB*: nuclear factor kappa B; *NLR*: nucleotide-binding oligomerization domain-like receptor; *NLRP*: NOD-like receptor thermal protein domain-associated protein**;** *Omp*: outer membrane protein; *PGN*: peptidoglycan; *STING*: stimulator of interferon genes; *TLR*: toll-like receptor. Created with BioRender.com.

During bacterial colonization of tissues, either as microbiota or infection, BEVs may be shed directly by pathogens or EVs by pathogen-infected cells^[215]^. BEVs carry microbial- or pathogen-associated molecular patterns (MAMPs/PAMPs) that can be recognized by pattern recognition receptors (PRRs) on the surface and in the cytoplasm of immune and non-immune cells^[14,216,217]^ [Figure 3]. The interaction of these MAMPs/PAMPs, which could be located on the external surface (LPS, Omp, lipoteichoic acid) or inside the vesicle (peptidoglycan, DNA, RNA, toxins) [Figure 1], and PRRs activate signaling pathways leading to the release of pro-inflammatory cytokines that trigger inflammatory responses for host defense^[14,209,217,218]^ [Figure 3].

For example, LPS, adhesins, and other virulence factors in the periodontal bacterium *Fusobacterium nucleatum-*derived EVs interact with Toll-like receptor (TLR)-4 and induce the expression of IL-8 and TNF-α in patients with inflammatory bowel disease (IBD)^[219]^. In contrast, *M. tuberculosis* and *M. bovis* EVs stimulate the release of IL-1β, IL-6, IL-10, IL-12, TNF, CXCL1, and MIP-1α in macrophages through TLR2 signaling^[220,221]^. LPS, lipoproteins, flagellin, and DNA carried by OMVs can interact with TLRs on microglia and macrophages to induce the release of TNF-α and IL-10^[222,223]^. DNA, RNA, and peptidoglycan cargo in *S. aureus* EVs enhanced the release of cytokines and chemokines by epithelial cells by activating several TLRs and nucleotide-binding oligomerization domain (NOD) 2^[224]^. Through the MyD88-dependent TLR4 signaling pathway, LPS carried by *P. aeruginosa* OMVs stimulates an inflammatory response in lung epithelial cells^[225]^. BEVs from *H. pylori, P. aeruginosa*, *N. gonorrhoeae*, and *C. jejuni* also induced the secretion of antimicrobial peptides, including human β-defensins and peptide LL-37, by human gastric epithelial cells^[183]^. LPS delivered into the cytosol by OMVs activate caspase-II mediated lytic cell death (pyroptosis)^[200]^. OMVs can deliver LPS in an array of human cells, including HeLa cells, bone marrow-derived macrophages, and THP1 macrophages, as well as mouse peritoneal resident cells through endocytosis^[200]^.

### BEV-induced inflammatory responses at the feto-maternal interface (placental tissues), resulting in adverse pregnancy outcomes

Intra-amniotic injections of Group B *Streptococcus* (GBS) EVs promoted the upregulation of pro-inflammatory cytokines and inflammation similar to the features of chorioamnionitis and induced apoptosis in the choriodecidual tissue^[226]^. This GBS EV-induced inflammation at the feto-maternal interface also promoted preterm birth and intrauterine fetal demise in mouse models^[226]^. Furthermore, treatment of decidua and placental cells with low dose *E. coli-*derived BEVs induced increased IL-6 levels, whereas a high dose was cytotoxic after 24 h of treatment^[26]^. Therefore, we reviewed the potential contributions of BEVs during pregnancy, highlighting their origins and mechanisms employed to maintain or disrupt feto-maternal immune tolerance.

#### Microbial vesicles and their potential contributions during pregnancy

The controversy surrounding the presence of a microbiome in the placenta is somewhat mitigated, and the consensus has emerged that the intrauterine environment is rather sterile. However, our reporting of BEVs in the placenta^[26]^ and a recent report that amniotic fluid also contains BEVs^[227,228]^ suggest that amplification of microbial nucleic acid, and identification of microbial antigens and other cellular fragments are more likely the confirmation of microbial vesicles than the presence of microbe itself. Amplification of placental BEVs and lack of any microbiome beyond noise levels expected from procedural aspects of experiments confirm that placental microbiome is a mistaken identity. The world of microbial vesicles in a sterile environment of pregnancy opens a plethora of questions and may answer several mysteries of pregnancy maintenance, immune homeostasis, microbiome formation in the fetus, and fetal immune privileges to commensal bacteria in utero, at birth, and during the early developmental stages.

#### Maternal gut microbiota-derived EVs can cross the placental barrier

Although the role of EVs in human pregnancy has been reviewed extensively^[35,36,229,230]^, data on microbial EVs in healthy pregnancies are still limited. However, maternal gut microbiota-derived EVs can cross the placental barrier to reach the fetus^[227]^. This EV-mediated in utero communication between the fetus and the mother was evidenced by the presence of bacterial RNA and proteins in the amniotic fluid of pregnant women^[227]^. The EVs derived from both compartments shared similar bacterial composition and protein cargo. This EV-mediated interaction may be required for priming the fetal immune system, which is essential for neonatal gut colonization^[227]^. A mouse model also reproduced these findings^[227]^.

This report is an extension of our previous study that identified BEVs in placental tissues^[26]^ and the findings of Nunzi *et al.*, who reported the presence of BEVs in human amniotic fluid via 16S-rRNA gene sequencing^[228]^. Furthermore, our study^[26]^ and that of Kaisanlahti *et al.* opined that the presence of BEVs in placental tissues or fetal compartments, irrespective of the source, is crucial for fetal immune priming, perhaps through low-grade immune stimulation^[227]^. The point at which this ‘beneficial’ immune stimulation becomes deleterious and the mechanism(s) that drive such phenotype require further investigation. Future years will generate evidence that BEVs have a significant role in priming human immune system development, and immune system development starts in utero and not during the early stages of development after birth in response to environmental microbial exposures.

### EV-DNA and its role in placental inflammatory response

EV-DNA is considered a mediator of cellular homeostasis^[231]^ as well as innate and adaptive immune responses^[79,232]^. Cellular excretion of potentially harmful damaged DNA prevents the induction of apoptosis and promotes survival^[231]^. Active release of EV-DNA, as indicated by increased concentrations of circulating DNA, correlates with the proportion of cells preparing for division (G1 phase)^[233]^.

**Figure 4:**
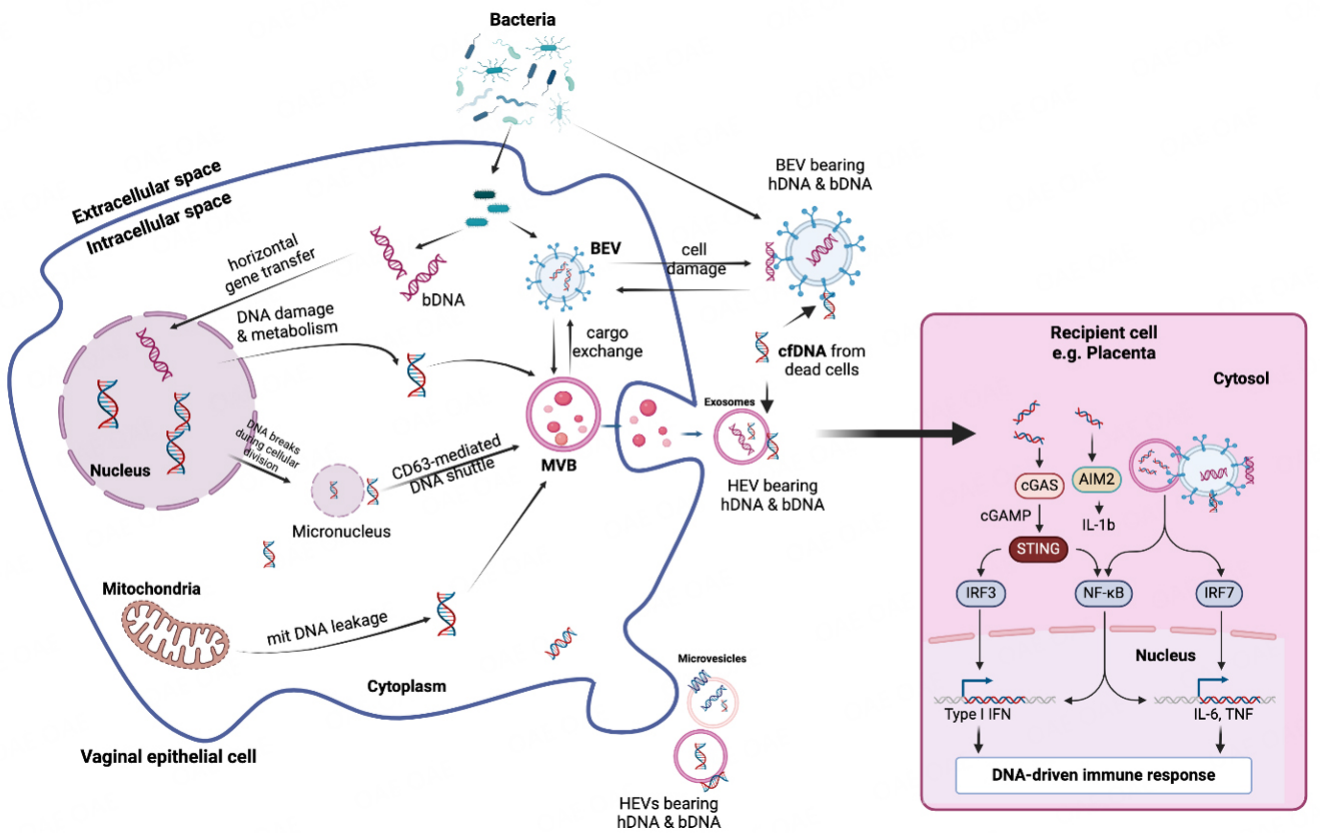
DNA-driven immune response mediated by DNA exchange between human and bacterial extracellular vesicles. Human/bacterial genomic/mitochondrial DNA carried by EVs can be delivered to cells as damage- (DAMP) or pathogen-associated molecular pattern (PAMP). EV-DNA can activate inflammatory pathways through cytosolic DNA receptors such as cGAS (cyclic GMP-AMP) and AIM2 (absent in melanoma 2 or interferon-inducible protein). Activation of the cGAS/STING pathway causes downstream release of type I interferons (IFN-α, IFN-β, IFN-ε). Activation of AIM2 produces interleukins and tumor necrosis factor (TNF). This may manifest as a subclinical sterile inflammatory response in gestational tissues such as the placenta and amniochorion. bDNA: bacterial DNA; BEV: bacterial extracellular vesicles; cfDNA: cell-free DNA; DNA: human DNA; HEV: human extracellular vesicles; IRF: interferon regulatory factor; mtDNA: mitochondrial DNA; MVB: multivesicular bodies; NF-κB: nuclear factor-kappa B; STING: stimulator of interferon genes. Created with BioRender.com.

Although whether BEVs and human EVs found at the feto-maternal interface exchange cargos has not been established, we hypothesize that human cells in this compartment can take up BEVs. In contrast, BEVs can internalize human products such as DNA, RNA, proteins, metabolites, and lipids. For instance, the human cells may internalize BEVs, or human EVs may bind to LPS released by BEVs, presenting it to membrane PRRs or facilitating its access to the cytosol, where it can trigger an inflammatory response^[234]^ [Figure 4]. Uptake of *G. vaginalis* BEVs by vaginal epithelial cells leads to vaginolysin-mediated cytotoxicity and increased release of IL-8^[235,236]^.

There could also be HGT between EVs from both species or between EVs and host cell genomic and mtDNA^[105]^. OMVs can carry DNA into human cells and exchange it with the human DNA via HGT^[145]^. The altered/damaged DNA in EV and cfDNA (from dead cells) that stick to the surface of EVs could subsequently be taken up by a recipient cell in an autocrine or paracrine manner. The DNA can trigger several cytosolic DNA receptors, including cyclic GMP-AMP synthase (cGAS), leading to the downstream release of type 1 IFN through activation of stimulator of interferon genes (STING) and translocation of NF-kB and IRF3 to the nucleus^[237,238]^. The DNA can also trigger AIM2, producing interleukins^[106,108,238,239]^ [Figure 4]. Mitochondrial DNA can also stimulate the cGAS/STING pathway^[240]^. This EV-DNA-induced inflammation is part of innate immune responses and interaction with adaptive immunity, as seen in cancer and malaria parasite infection^[108]^.

#### Placenta inflammatory response

It is plausible that the “sterile” inflammation that leads to adverse birth outcomes^[241,242]^ may be a product of chronic inflammatory response to damaged/altered EV-DNA or other PAMPs/DAMPS (LPS, Hsp70, HMGB1) carried by human/BEVs^[13,30,69,77,243-250]^, and delivered to the placenta. cfDNA can stick to EVs, be internalized by cells, and induce several inflammatory responses mediated by TLRs and other nucleic acid receptors through the mechanisms described in Figures 3 and 4^[106,251]^.

We postulate that in gestational tissues such as the placenta and amniochorion, EV-DNA from pathogenic bacteria (due to maternal infections such as periodontitis, BV, urinary tract infection, sexually transmitted diseases during pregnancy) can be delivered to the cytosol and activate the cGAS/STING and AIM2 signaling pathways to release type I IFN and interleukins [Figures 3 and 4]. Because there may be no clinical infection by any pathogen, this may manifest as subclinical sterile inflammation that can induce adverse reproductive outcomes.

Placental inflammatory responses that underpin maternal-fetal allograft rejection, preeclampsia, intrauterine growth restriction (IUGR), and preterm birth are induced by infectious and sterile stimuli^[242]^. However, sterile intra-amniotic inflammation is more common than microbial-associated intra-amniotic inflammation^[250,252-254]^. Chronic placental inflammatory lesions are mediated by cytotoxic T cells and IFN-γ-inducible CXCR3 ligands^[255]^. Upon antigenic stimulation, T cells can secrete EVs carrying both gDNA and mtDNA that trigger IFN1 response from DCs via the cGAS/STING pathway. This primes the DCs against subsequent viral infections^[232]^. T cells’ local EV-DNA inflammatory activities can be explored further in the context of immune tolerance surveillance at the feto-maternal interface.

A considerable proportion of pregnant women with inflammation of gestational tissues, which eventually deliver prematurely, do not have an infection^[254,256,257]^. There can be innumerable reasons for a sterile inflammatory condition associated with premature delivery; however, recent findings on BEVs support the concept that maternal morbid or behavioral factors that may be linked to adverse events, may also be compounded by pathogenic microbial EVs, or subclinical infection in mothers may deliver BEVs to facilitate a sterile inflammatory condition. This sterile inflammation often mimics infectious inflammation seen during adverse pregnancies. In these instances, microbial culture or other molecular diagnostic approaches may render negative reports and mismanagement of a patient with a subclinical infection that sheds BEVs loaded with immunogenic factors. Therefore, the possible role of EV-DNA, as well as other alarmins carried by EVs, in chronic inflammation of gestational tissues that lead to adverse outcomes requires more investigation. Moreover, the threshold at which BEVs' proposed immune priming role in gestational tissues such as the placenta and fetal membranes changes to an overt labor-inducing inflammatory response needs to be determined along with the underpinning mechanisms. EV-DNA could be used for priming the immune system to defend against subsequent infection^[108]^. However, this is yet to be demonstrated in the fetus *in utero*.

### Benefits and disadvantages of EV-mediated responses at the feto-maternal interface (placental tissues)

#### Priming of the fetal immune system

**Figure 5:**
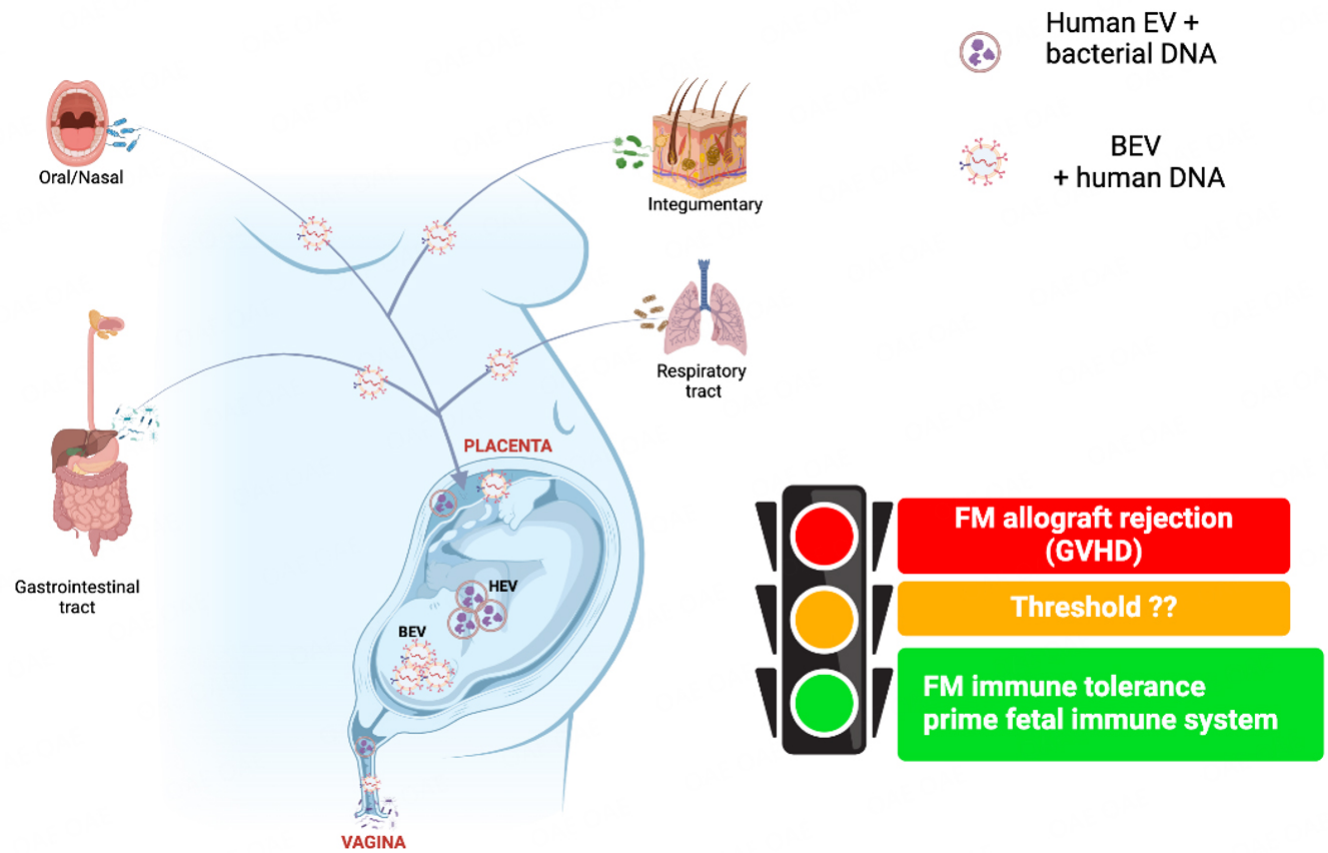
Extracellular vesicle-mediated feto-maternal immune tolerance. The feto-maternal immune tolerance that maintains normal pregnancy is partly mediated by BEVs from various microbiotas. Maternal BEVs can also carry DAMPs/PAMPs to the feto-maternal interface (placenta tissues) and trigger adverse immune responses that may manifest as feto-maternal allograft rejection in the form of graft-versus-host disease (GVHD). However, the threshold at which such a switch occurs, and the mechanisms involved are yet to be determined. Conversely, pathogenic bacteria can shed BEVs that can cause inflammatory responses often associated with adverse pregnancy conditions. BEV: bacterial extracellular vesicle; DAMPs/PAMPs: damage- and pathogen-associated molecular patterns; FM: feto-maternal; HEV: human extracellular vesicle. Created with BioRender.com.

Commensal bacteria such as lactobacilli can produce BEVs that stimulate innate immune responses to control infection by pathogens. This immunostimulation may prime the host cells to mount an adequate immune response against foreign invaders upon subsequent exposure^[209]^. However, in uncomplicated pregnancies, the most prevalent BEVs resident in the placenta and fetal membranes and their origins [Figure 5] are yet to be established. Nevertheless, the presence of BEVs from health-promoting lactobacilli at the feto-maternal interface may be recognized as “self-antigens” or, depending on their abundance/load, could prime the fetal immune system against infection and prevent infection-associated pro-inflammatory responses that precede labor and preterm birth. Because ascension from the lower genital tract is the primary pathway for intra-amniotic bacterial colonization^[258]^, we suspect that the proposed immune priming effect^[26,227]^ may be partly mediated by lactobacilli BEVs. This concept is supported by the report that BEVs from common vaginal lactobacilli carrying numerous proteins and metabolites protected human tissues from HIV-1 infection^[2]^. Therefore, the genome, proteome, and metabolome of *Lactobacillus*-derived EVs can be analyzed to determine if the antimicrobial and anti-inflammatory properties of *Lactobacillus* spp. such as *L. crispatus,* which are mainly linked to healthy vaginal microbiota and term delivery^[22,259-261]^, are exhibited by their EVs independently, and whether such BEVs are *bona fide* residents of the placenta and fetal membranes. This can be compared to the EV cargo of BV/preterm birth-associated bacterial species such as *Gardnerella vaginalis,* GBS, and *Ureaplasma* spp. commonly isolated from the intrauterine/intra-amniotic cavity^[236,258,262-267]^.

Furthermore, the *Ureaplasma* DNA load seen in amniotic fluid^[268-278]^ may have been transported by EVs, and this may not necessarily indicate pathogenesis. However, molecular diagnostic strategies such as polymerase chain reaction and 16S gene sequencing employed in those studies may have omitted bacterial DNA packaged in EVs with pathogenic or immune priming potentials. If *Ureaplasma* spp., *G. vaginalis*, and GBS can find their way to the amniotic fluid, they may continue to shed EVs to enhance inflammatory pathologies. So, even if the bacterial load is less, thousands of BEVs shed can cause damage and may be resistant to antimicrobial treatment. Therefore, controlling inflammation becomes of utmost importance in reducing adverse incidences compared to mere antibiotic treatment. Additionally, because the most prevalent BEVs in gestational tissues at a given time point may not be of vaginal origin, the investigation needs to be expanded to BEVs from other niches in the body and external environment.

Suppose the immune priming action of placental BEVs is confirmed. In that case, such BEVs can be explored for vaccine development and therapeutic purposes^[279,280]^ [Table 3] to either stimulate or suppress feto-maternal immune responses against intrauterine infection or microbial invasion of the amniotic cavity that cause preterm premature rupture of membranes (PPROM) and preterm labor^[258,265-267,285]^. This is because, rather than aiding pathogenesis, exosomes and OMVs are potent immune modulators^[286]^, as demonstrated in pertussis (whooping cough) caused by *Bordetella pertussis*^[287]^. Furthermore, *S. aureus* EVs engineered to serve as vaccine candidates elicited adaptive immune response and conferred protection against lethal sepsis caused by *S. aureus* in mice^[133]^. Additionally, *S. pneumoniae-*derived EVs incubated with murine DCs were rapidly internalized and promoted the release of TNF-α, which constitutes the inflammatory response^[288]^.

**Table 3:** Summary of usage and applications of bacterial and human extracellular vesicles and future aspects for exploration

**Use/application**	**Extracellular vesicle**	**Feature/summary of use**	**References**
Immune priming	BEV	● Antigen-presenting and immune stimulation ● Recognized as self-antigen	[26,227,228]
	HEV	● EVs from APCs carry p-MHC-II and costimulatory signals, and directly present the peptide antigen to specific T cells to induce their activation ● Placental EVs mediate immune tolerance during gestation	[230,281]
Vaccine and adjuvant development	BEV	● Present multiple antigens simultaneously in a native state to elicit effective immune responses ● Stimulate immune responses against bacterial and viral infections ● Examples: BEVs from *N. meningitidis*, *V. cholerae*, *B. pertussis*, *S. aureus*, *S. pneumoniae*, *C. perfringes*, *B. anthracis*, *etc.* ● Engineered OMVs as adjuvant-free vaccine platform for important pathogens	[2,4,25,209,282,279,280,151]
	HEV	● EVs from macrophages and DCs against *M. tuberculosis* and *T. gondii* infections ● EVs are also used to modulate immune response against tumor development	[44]
Therapeutics and drug delivery	BEV	● Carry and deliver several bioactive molecules ● Enter distant organs from the circulation ● Non-replicating and great biostability ● Easy to modify by electroporation ● Treatment of gut, brain, bone diseases and cancers	[209,216,283]
	HEV	● EVs can be packed with small molecule drugs to evade immune surveillance and delivered intact and directly to the target tissue ● MSC-derived EVs promote wound healing via Wnt-β catenin pathways and used in the treatment of cardiovascular, lung, renal, and liver diseases, GVHD, *etc*.	[44,281,284]
Biomarkers	BEV	● Readily found in body fluids ● Diagnosis of bacterial infections, lung disease, bone disease, colorectal cancer, and pancreatic adenocarcinoma	[25,283]
	HEV	● Widely distributed and more readily available through liquid biopsies using blood, saliva, urine, breast milk, sperm, CSF, and vaginal fluid ● Contain DNA, miRNA, and protein biomarkers ● Applied in the diagnosis of cancer, asthma, COPD, GVHD, COVID-19 infection, etc.	[44,105,106,108,281]
Future aspects for exploration	BEV	● Address which receptors determine the uptake of BEVs by host cells ● Mechanism of cargo packaging ● Address the challenge of LPS-associated biosafety when Gram-negative BEVs are used as vaccines or drug delivery vehicles ● Gram-positive BEVs may be a better choice for drug delivery ● Identify a faster, cost-effective and efficient (high yield) isolation method from a wide range of biological specimens ● Identify unique (surface) markers present on BEVs from different sources	[2,283]
		Placenta and fetal membranes: ● Identify the most prevalent BEVs ● Targeting BEVs specifically to these tissues ● Do BEVs in these tissues differ between term *vs.* preterm deliveries or PE *vs.* normotensive pregnancies? ● Do BEVs from placenta elicit similar immune responses in other gestational tissues ● Do BEVs from BV/PTB-associated bacteria elicit inflammation in gestational tissues?	
	HEV	● Identify mechanisms responsible for the uptake of EVs containing DNA ● Identify mechanisms for DNA packaging in EVs ● Standardization of EV-DNA isolation and analysis techniques ● Resolution of differences in EV-DNA distribution, localization, and structure for diagnostic and functional studies	
		Placenta and fetal membranes: ● Are there human cells/EVs containing bacterial EVs or products in gestational tissues? ● Do bacterial cells contain human EVs in gestational tissues?	

APCs: antigen-presenting cells; BEV: bacterial extracellular vesicles; BV: bacterial vaginosis; COPD: chronic obstructive pulmonary disease; COVID-19: coronavirus disease 2019; CSF: cerebrospinal fluid; DCs: dendritic cells; EV: extracellular vesicles; GVHD: graft-versus-host disease; HEV: human extracellular vesicles; LPS: lipopolysaccharide; MSC: mesenchymal stem cell; PE: preeclampsia; p-MHC-I: major histocompatibility complex II with antigenic peptide (p); PTB: premature birth; Wnt-β: wingless-related integration site-beta.

#### Stimulation of preeclampsia, preterm labor and birth

The proposed priming action of BEVs in the feto-maternal interface is adjudged to be mediated by a low-grade inflammatory stimulation insufficient to induce labor^[26]^. Pregnancy is characterized by low-grade systemic inflammation and immune tolerance that allow implantation and placentation^[255,289]^. Disruption of this physiologic inflammatory state could lead to a breakdown in fetal-maternal tolerance and uncontrolled pro-inflammatory responses that trigger labor prematurely or other undesired reproductive outcomes such as preeclampsia and IUGR^[255,290]^. For instance, intraperitoneal injection of fetal DNA or CpG, which eukaryotic/BEVs can transport to uterine tissues or feto-maternal interface [Figures 3-5], induced TLR-9, STING, and NF-κB-mediated inflammatory release of IL-6, leading to fetal resorption, preeclampsia, preterm labor, and preterm birth in mice with additional immune impairment^[291-294]^. The BEV-associated immune stimulation must remain optimal throughout gestation, or an overt EV-mediated inflammatory response with deleterious effects may ensue without any identified pathogen [Figures 4 and 5].

What is yet unknown is the threshold at which preeclampsia- or labor-associated pro-inflammatory responses can be triggered by BEVs in placental tissues. We speculate that, like an increase in bacterial load or altered microbiota, an increase in placental BEVs and their virulence factors carried as cargos beyond the tolerable/immune priming threshold may induce an overt inflammatory response that could lead to premature expulsion of the fetus or pregnancy complications such as preeclampsia and IUGR or neonatal complications^[230]^. Such immune response could manifest as acute placental inflammation (i.e., maternal and fetal inflammatory responses)^[242,295-298]^ or chronic placental inflammation^[255,299]^. If determined, this will improve our understanding of the concept of "sterile inflammation", which could be induced by human/BEV that may have exchanged immunogenic cargo, perhaps at a site distant from the placenta or fetal membranes, compared to infectious inflammation. It could also shed more light on the poor performance of antibiotics in reducing the incidence of preterm birth^[300,301]^. The mechanisms that propagate subclinical inflammation even after clearance of the infectious stimuli may be associated with BEVs and their virulence factors transported by human cells/EVs that help them evade immune clearance. For example, BEVs from *S. aureus* strains that cause severe inflammatory diseases in humans and animals contain a core proteome enriched with virulence factors. The BEV core proteome predicted the *S. aureus* strain and severity of infection, ironically in the absence of the pathogen^[153]^.

#### Novel preeclampsia or preterm birth-associated inflammatory biomarker targets and preventive/therapeutic interventions

EV-DNA has been speculated to be more beneficial than plasma cfDNA in cancer diagnosis using certain liquid biopsies^[302-304]^. Elevated serum BEV IgG antibody-based asthma and chronic obstructive pulmonary disease diagnosis have also been tested^[137]^. Additionally, metagenomic and metabolomic profiling of stool BEVs revealed significantly altered *Firmicutes* and *Proteobacteria* as well as amino acids, carboxylic acids, and short-chain to long-chain fatty acids metabolism in colorectal cancer patients^[305]^. Similarly, human EVs and BEVs found in placental tissues can facilitate the identification of new preeclampsia or preterm birth-associated inflammatory biomarker targets. Our group is currently exploring immune proteins packaged in EVs in the genital tract of preeclamptic women. As a follow-up from our recent study^[26]^, we can explore whether placental BEV composition varies between preeclamptic and normotensive, as well as preterm-delivered and term women using metagenomics, proteomics, and metabolomics profiles of such pregnant women. We could also explore whether bacterial cells (not BEVs) package human EVs and the effect of BEVs on placental tissues *in vitro* or *ex vivo*.

Meanwhile, human EV-derived inflammatory proteins, miRNAs, and lipids that are associated with placental dysfunction, preterm labor, and birth have also been identified in maternal plasma across different gestational time points^[35,306,307]^. Moreover, after vaginal infection with *E. coli*, intravenous IL-10 encapsulated in exosomes delayed preterm birth by reducing feto-maternal uterine immune cell inflammation^[308]^. These reports indicate great promise for the utility of EVs as early predictors for preterm birth and candidates for safe and efficient drug delivery to the placenta and fetal membranes [Table 3]. However, these reports are primarily on human EVs, whereas data on BEVs are still minimal.

It is plausible that the drivers of placental inflammatory responses could be culpable for the disruptive phenotypes. This is because, even in the absence of clinical infection, PAMPs/DAMPs carried by and exchanged between human and BEVs can be transferred to placental tissues, gain access to the amniotic cavity, and trigger fetal inflammatory response, leading to maternal anti-fetal rejection^[242,255]^. For example, *B. fragilis* OMVs (without the bacteria) activated a broader range of host innate immune receptors (TLR2, TLR4, LR7, and NOD) compared to their parent bacteria (TLR2 alone) due to their enrichment with RNA and peptidoglycan cargo and their ability to transport this cargo directly into host epithelial cells^[309]^. Though this was demonstrated in intestinal epithelial cells, *B. fragilis* is a *bona fide* member of the vaginal microbiota as well^[21,22]^. Therefore, it is possible for OMVs released by this species to transport their cargo to the placenta, either hematogenous or through the vagina, and stimulate what may seem like a sterile inflammatory response.

The EVs and their inflammatory cargo could also emanate from the fetal cells and be delivered to maternal gestational tissues^[310]^. So, there could be bidirectional trafficking of inflammatory human EVs (and possibly BEVs) between the mother and fetus^[29,30,230]^. This is a precursor for inflammation-associated placental dysfunction, preeclampsia, fetal growth restriction, spontaneous preterm labor and postnatal developmental impairments^[35,230,311-314]^.

*S. aureus-*derived BEVs that contain proteins involved in metal ion acquisition may compete with bacterial and host cells, depriving them of such nutrients and thereby inhibiting their growth and survival^[153]^. This may be particularly beneficial in preventing the colonization of gestational tissues by BV and preterm birth-associated *G. vaginalis* that acquire iron from host cells for survival^[315-317]^. Moreover, Gram-positive bacteria are projected to be better candidates for vaccine development than Gram-negative bacteria because they lack LPS^[152]^ [Table 3].

Consequently, the health-promoting low-grade inflammation induced by lactobacilli in the lower genital tract can be replicated in the feto-maternal interface using *Lactobacillus*-derived BEVs as vaccines targeting the placenta. It has been shown that *L. crispatus* and *L. gasseri* EVs protect human cells and tissues from HIV-1 infection by reducing the availability of gp120 to HIV-1 target cells. As expected, the lactobacilli-derived EVs contain numerous anti-HIV-1 bacterial metabolites and proteins^[318]^. BEV protein cargos from other vaginal commensals such as *S. aureus*, *G. vaginalis*, *Enterococcus faecium*, and *Enterococcus faecalis* have produced similar anti-HIV effects by steric hindrance or modification of gp120^[319]^. These data support our proposition of further proteomic and metabolomic analysis of lactobacilli BEVs *vs.* BV/preterm birth-associated BEVs for better comparative understanding.

## CONCLUDING REMARKS AND FUTURE PERSPECTIVES

As discussed in this review, host-bacteria interaction is facilitated by BEVs and not only through direct contact between human and bacterial cells. The EVs are released by both bacterial and human cells and transfer bioactive molecules that influence the activity of the recipient cells, which may be distant from the producing cells. BEVs package multiple PAMPs that mimic and sometimes surpass the immunogenic properties of the secreting bacteria^[42,320]^. That is, direct colonization of tissues by bacterial cells is not required for immunogenic stimulation. This phenomenon is important in the feto-maternal interface, where optimum tolerance between the mother and fetus is required until delivery at term (37-40 weeks). Though the sterility of the placenta is still debatable, BEVs from diverse sources have been identified in this tissue^[26]^ and the amniotic cavity^[227]^. These BEVs can be internalized by human cells, which may help them evade host immune clearance. Though it appears logical, whether bacterial cells internalize human EVs is yet to be determined. However, the presence of BEVs in placental tissues or the amniotic cavity is believed to trigger a low-grade immune response that primes the fetal immune system for ex-utero survival but is insufficient to disrupt the progression of pregnancy. This appears to be another mechanism that propagates feto-maternal immune tolerance, as observed in BEV-mediated maintenance of intestinal immune homeostasis^[216,321]^. However, because some pregnant women still experience inflammation-associated diseases such as preeclampsia and preterm birth without clinical infection, the point or dose at which the BEV-mediated low-grade inflammation develops into a suboptimal immunological response capable of disrupting the typical sequence of pregnancy needs to be elucidated. Furthermore, the mechanisms that drive such feto-maternal intolerance should enhance our understanding of EV-associated pregnancy complications.

On the beneficial side, BEV vaccines exhibit immunostimulatory efficiency akin to that of the inactivated whole-cell vaccine, stimulating both cell-mediated and humoral immune responses in animals^[150,322,323]^. BEV vaccines have also been applied to tackle meningococcal group B disease in humans^[324]^. OMV vaccines or adjuvants could be targeted at the placenta to prime the fetus against future exposure to harmful human/BEVs carrying PAMPs/DAMPs [Table 3].

Moreover, as demonstrated in the administration of *Ligilactobacillus animalis, Akkermancia muciniphila, Lactobacillus plantarum*, and *Proteus mirabilis* BEVs to treat bone disease, glucose intolerance, obesity, IBD, and stress-induced depression-like behaviors^[325-329]^, native/natural BEVs from commensal or probiotic Gram-positive bacteria such as lactobacilli (to avoid LPS action) could serve as potential therapeutic candidates to promote a eubiotic and anti-inflammatory state in the feto-maternal interface while keeping the intrauterine environment “sterile”. This requires a comprehensive omic analysis of lactobacilli-derived EVs in comparison with EVs from common vaginal pathogens. We could also determine whether placental BEVs differ between women who deliver at term without labor and those who deliver preterm.

In summary, the exchange of cargo between human and BEVs and the isolation of BEVs in placental tissues and amniotic cavity have revealed new perspectives on the pathologic mechanisms of inflammatory pregnancy complications. The presence of BEVs in the placenta primes the fetal immune system. BEVs in gestational tissues appear to be good candidates for predicting adverse reproductive outcomes, as well as for vaccine development and drug delivery during gestation. Future studies should determine whether bacterial cells take up human EVs, the diagnostic potential of placental BEVs, and the mechanistic link between placental BEVs and pregnancy complications such as preeclampsia and preterm labor and birth.

## DECLARATIONS

### Copyright

© The Author(s) 2024.
